# Supplementary figures and images for: Proximity Labelling Reveals the Compartmental Proteome of Murine Sensory Neurons
Source: Eur J Pain. 2026 Apr 26;30:e70277. doi: 10.1002/ejp.70277 (PMC13111903; doi:10.1002/ejp.70277)

A

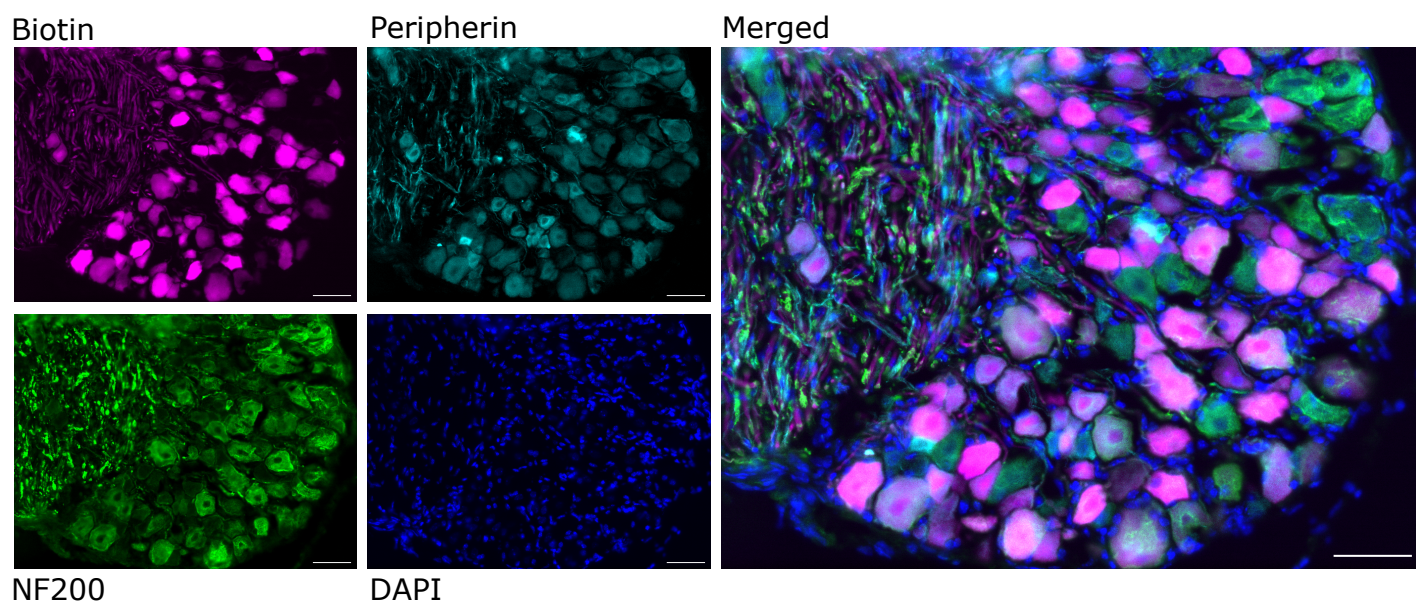

B

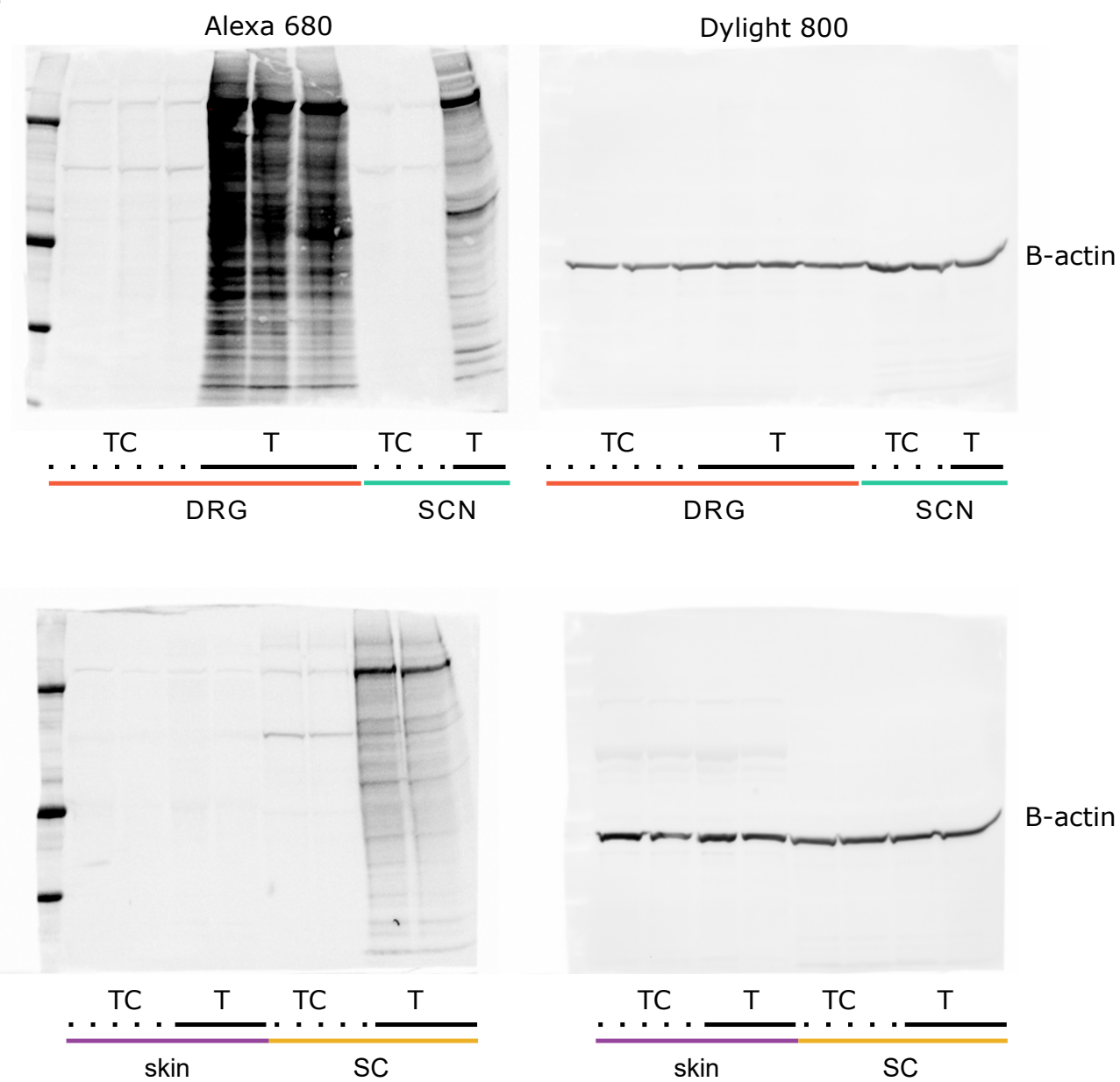

Supplement: Supplementary file 1 — Figure S1: A. Immunohistochemistry of AdvCre;TurboIDfl/fl with sensory neuron markers peripherin and NF200. Scale bar = 50 μm. B. Western Blot analyses for TurboID (left) and B‐actin loading controls (right). AdvCre;TurboIDfl/fl (T) and TurboIDfl/fl (TC) samples for each tissue were loaded. DRG, dorsal root ganglia; SCN, sciatic nerve; SC, lumbar spinal cord. [file EJP-30-0-s002.pdf]

A

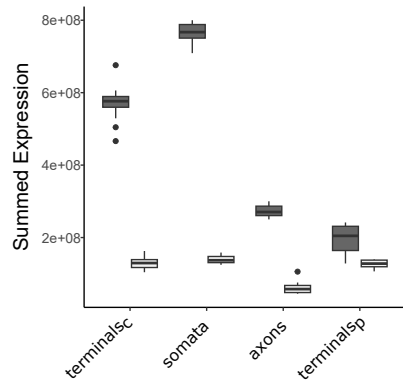

B

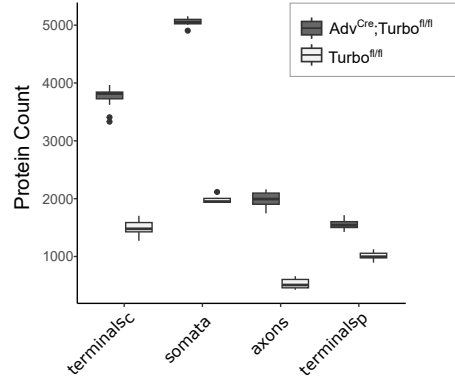

C

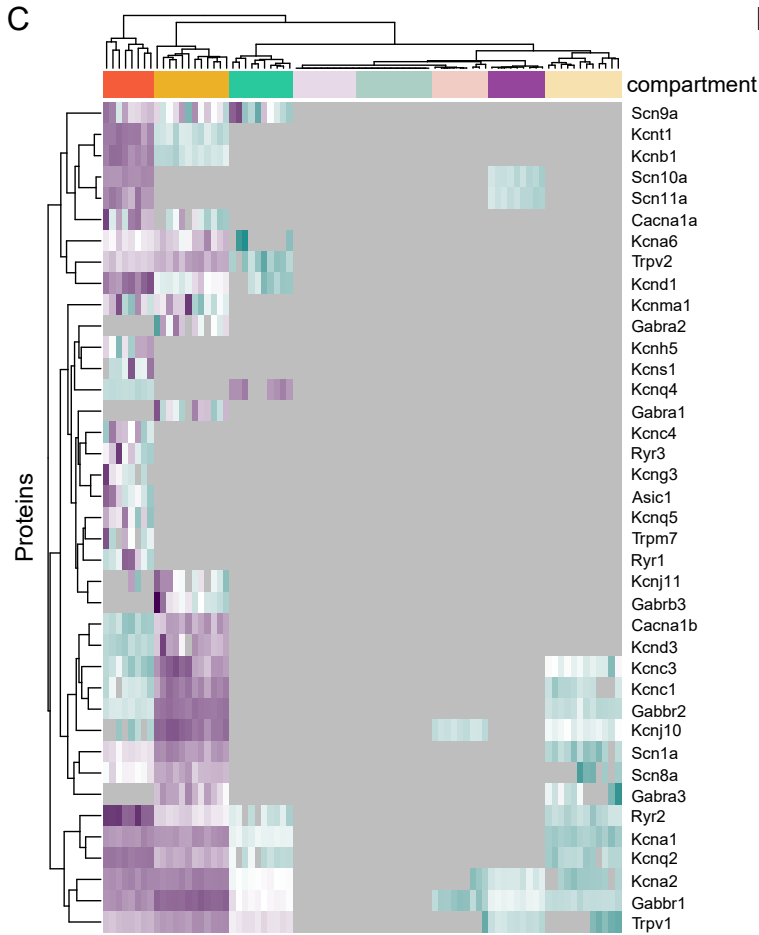

D

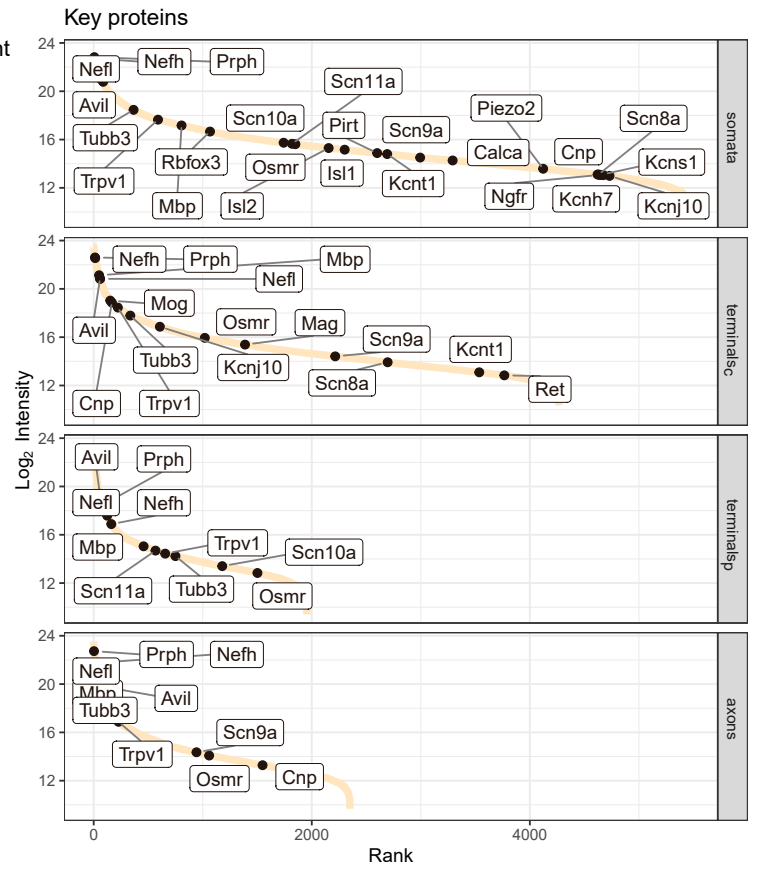

Supplement: Supplementary file 2 — Figure S2: DIA‐PASEF mass spectrometry of AdvCre;TurboIDfl/fl and TurboIDfl/fl control samples across sensory neuron compartments. (A, B) Summed expression (A), and Protein Group counts (B) by tissue and TurboID status. C. Heatmap of ion channel expression across all samples. D. Dynamic range for proteins of interest in AdvCre;TurboIDfl/fl samples plotted as expression (log2 Intensity) per component. terminalsp = peripheral terminals (skin); terminalsc = central terminals (SC). Relates to Figure 2. [file EJP-30-0-s001.pdf]

A

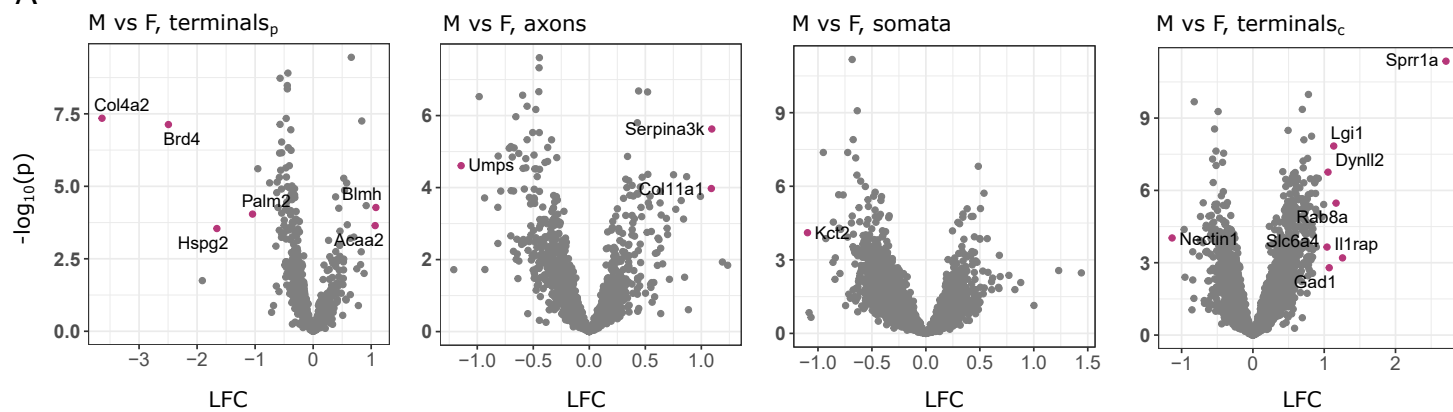

B

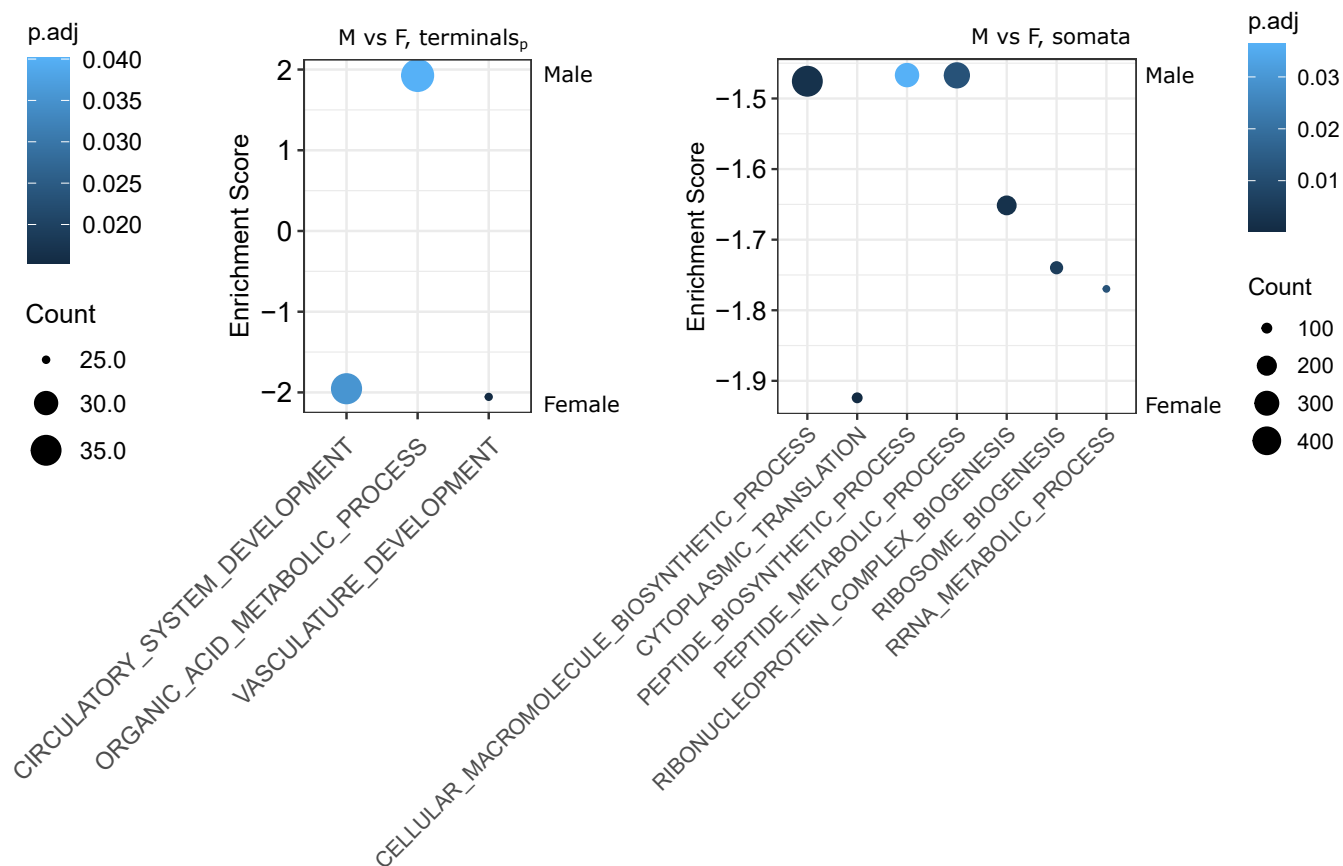

Supplement: Supplementary file 3 — Figure S3: Sexual dimorphism across neuronal compartments. A. Differential protein expression by compartment, comparing male vs female mice within TurboID‐enriched proteomes. Significant proteins are in pink. LFC = log2 fold change B. Biological Pathway analysis for peripheral terminals and DRG on ranked fold changes between male and female samples. Counts reflect the number of proteins in each respective term. terminalsp = peripheral terminals; terminalsc = central terminals. [file EJP-30-0-s013.pdf]

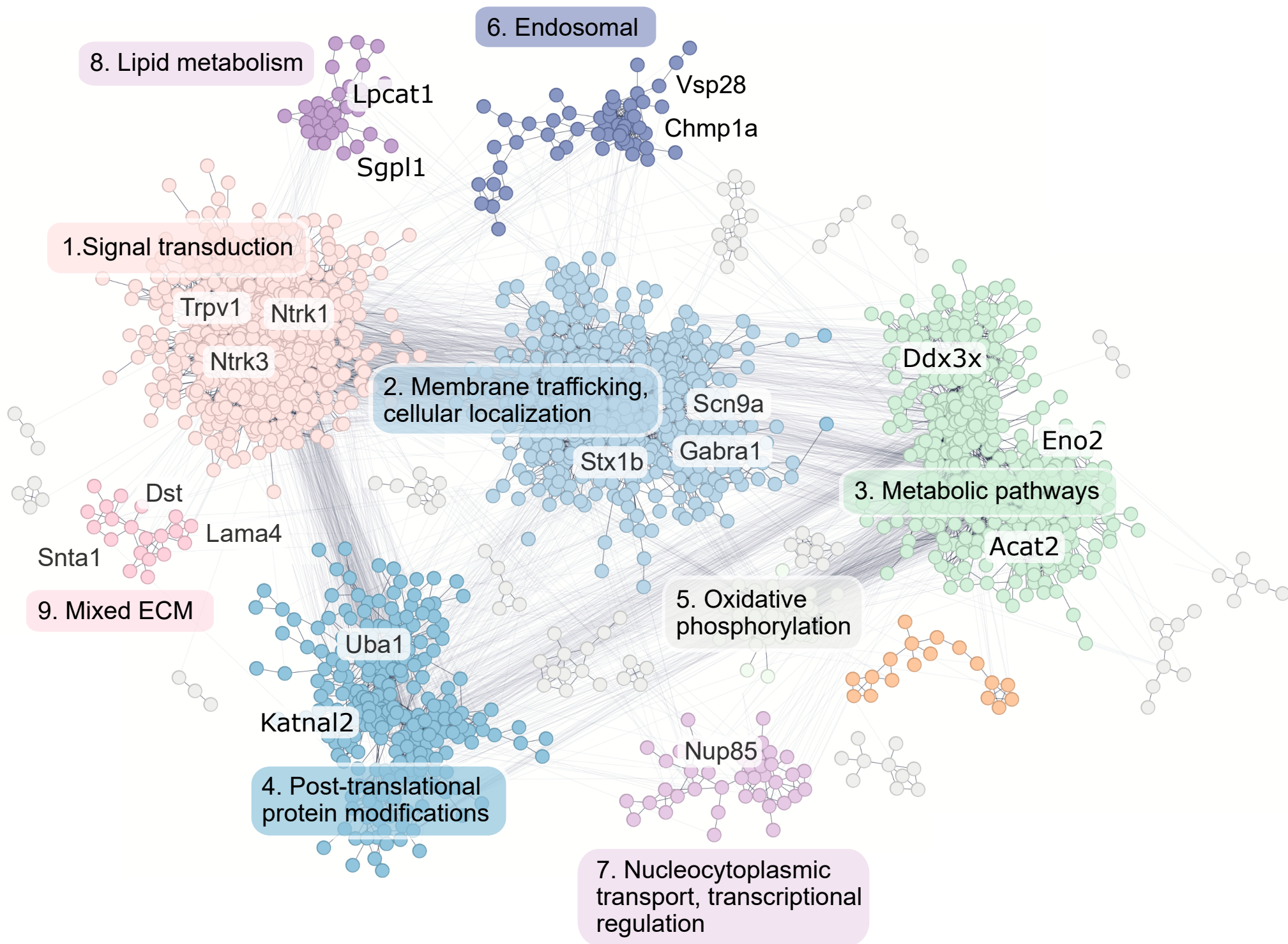

Supplement: Supplementary file 4 — Figure S4: Community clustering of the TurboID‐enriched central terminal proteome, with edges denoting high confidence protein‐protein interactions. Protein nodes are coloured by cluster, and clusters are described by functional summaries with proteins of interest highlighted. Full functional enrichments are available in Table S8. [file EJP-30-0-s021.pdf]

A

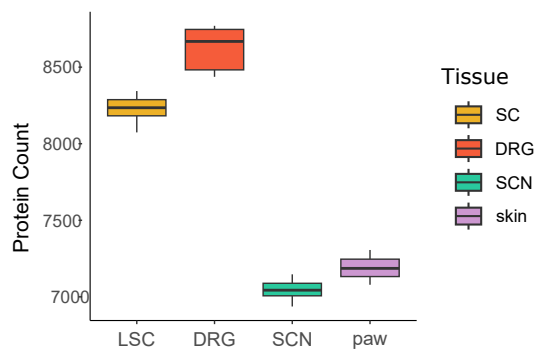

B

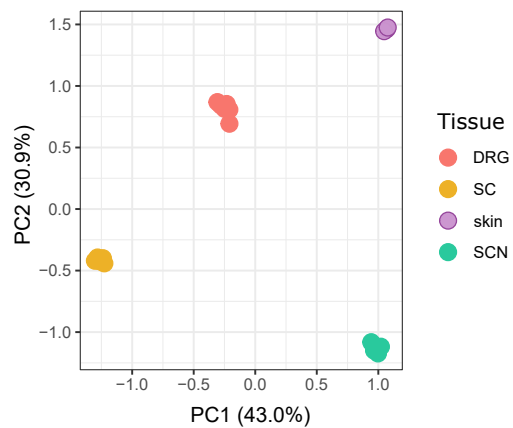

C

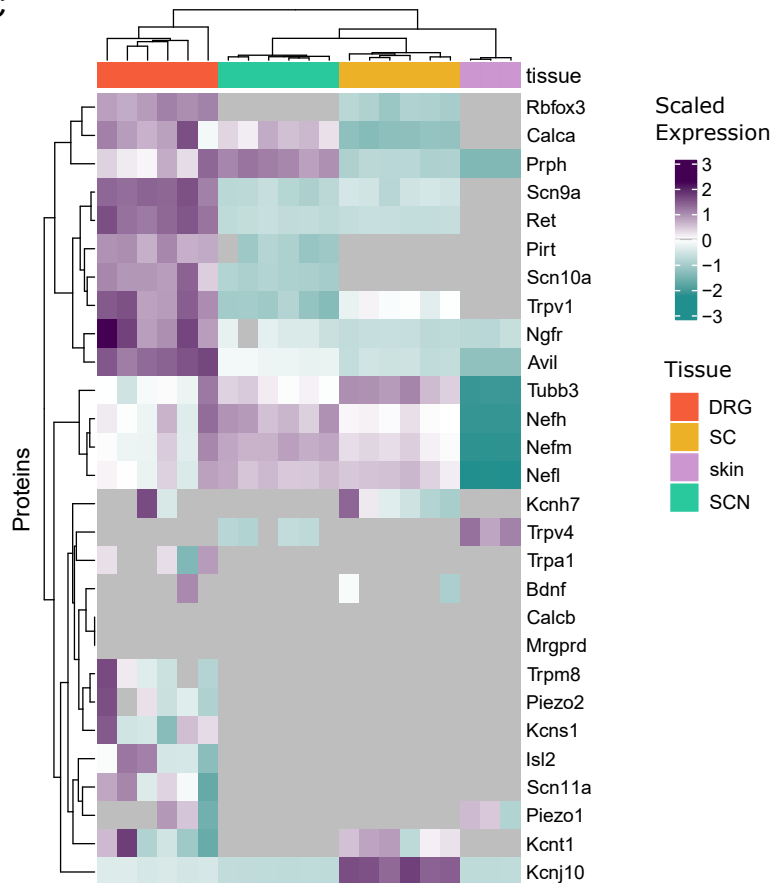

D

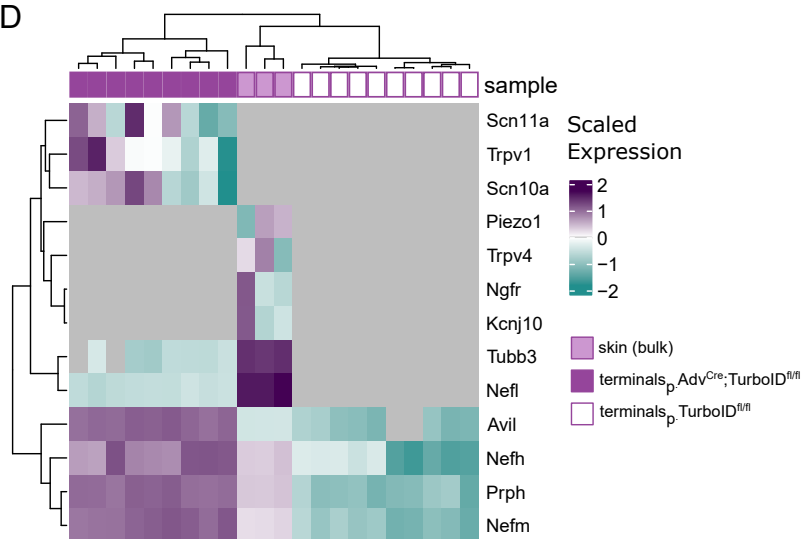

E

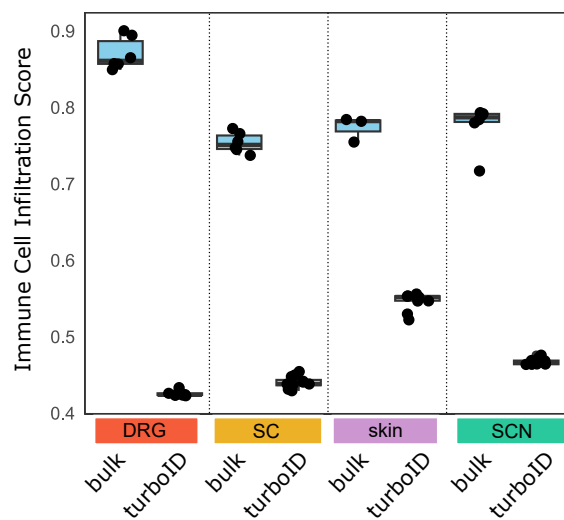

Supplement: Supplementary file 5 — Figure S5: Bulk proteomes of the four analysed tissues from wildtype mice. A. Protein Group (PG) counts per tissue. B. PCA by tissue. C. Heatmap of the scaled expression of selected key neuronal genes. D. Comparison of key neuronal proteins between peripheral terminals of AdvCre;TurboIDfl/fl, those of TurboIDfl/fl, and bulk proteomes obtained from skin samples. E. Depletion of ImmuCellAI immune cell signatures is evident in AdvCre;Turbofl/fl samples compared to corresponding bulk samples from wildtype tissues. [file EJP-30-0-s011.pdf]

A

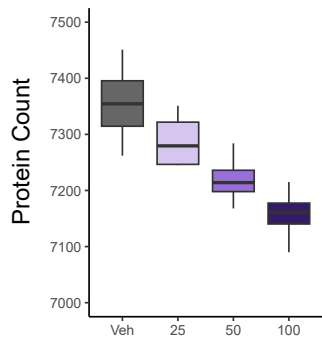

B

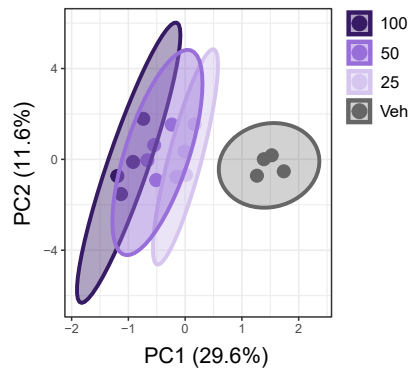

C

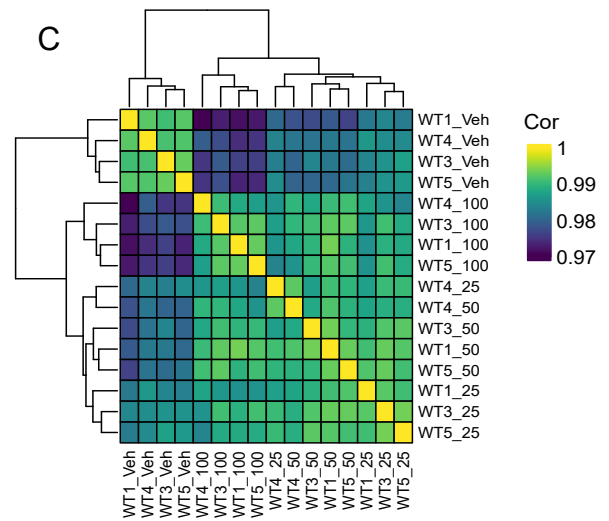

D

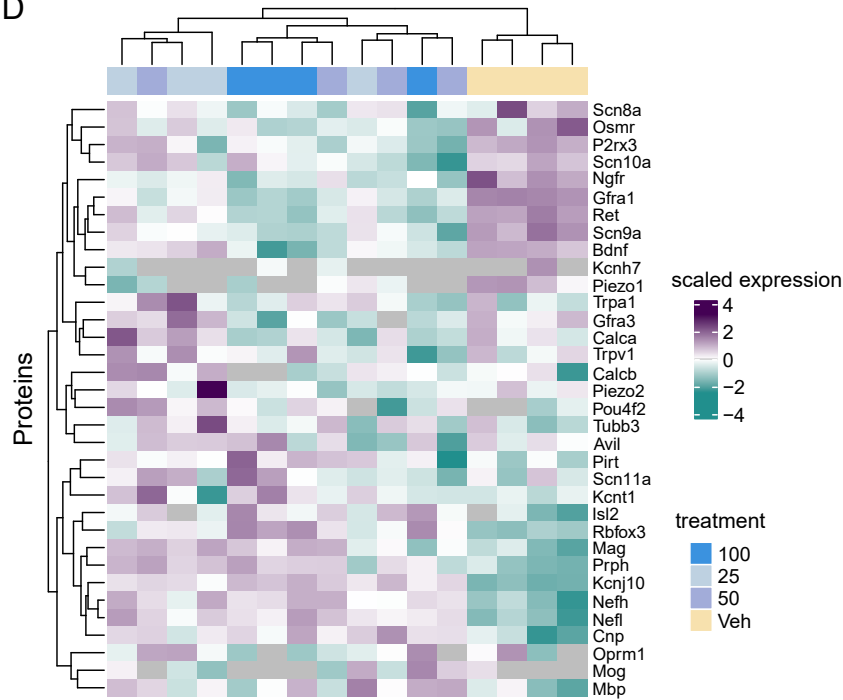

E

Oxaliplatin vs vehicle, bulk proteomics

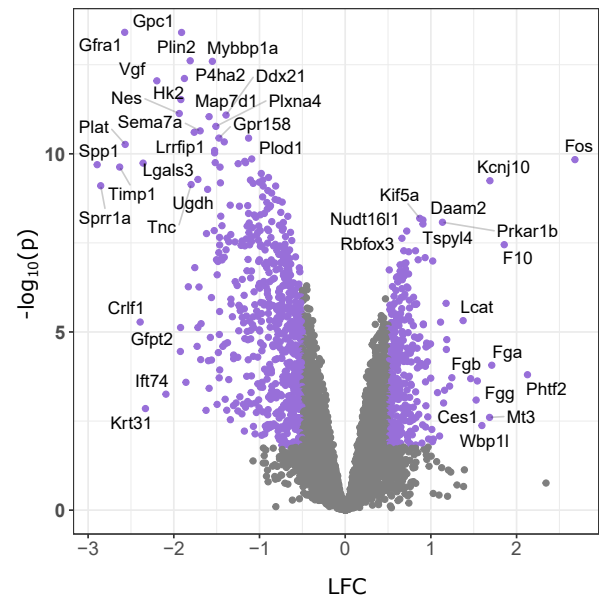

F

DEPs, vs Yang et al 2022.

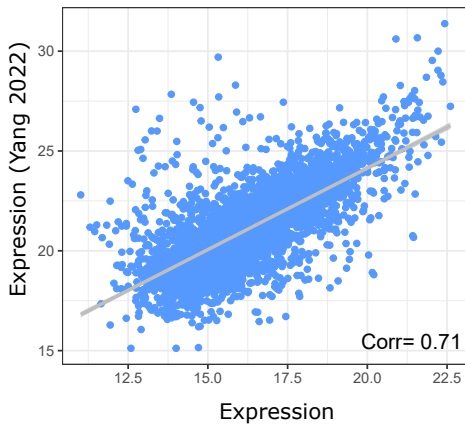

G

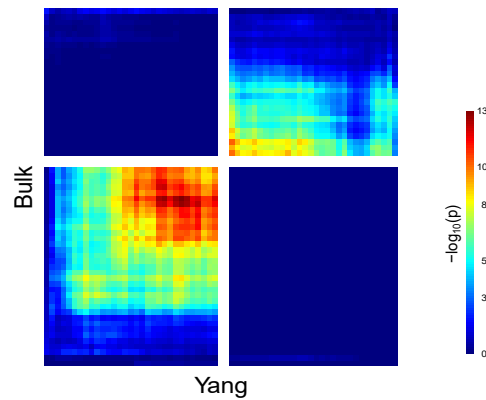

Supplement: Supplementary file 6 — Figure S6: Bulk Proteomics of DRG explants exposed to oxaliplatin (25, 50 or 100 μM) or vehicle (Veh). A. Protein counts. B. PCA across sample conditions shows clear separation of oxaliplatin‐treated samples versus Veh‐treatment. C. Correlation across samples, labelled as oxaliplatin (25, 50, 100) or vehicle (Veh). D. Heatmap across selected key neuronal proteins. E. Differentially expressed proteins (DEPs) between 100 uM oxaliplatin‐ and Vehicle‐treated DRG explants using bulk proteomics. Purple dots indicate significantly regulated proteins (FDR < 0.05, LFC > 0.5). F. Correlation of relative protein abundance between our DEP dataset (from E) with previously published proteome data from in vivo oxaliplatin‐treated DRG (Yang et al. 2022). G. Rank‐rank hypergeometric overlap between our DEP dataset (in E) and DEPs of the previously published oxaliplatin‐treated proteome (Yang et al. 2022). DEP, differentially expressed protein; LFC, log2 fold change. [file EJP-30-0-s008.pdf]

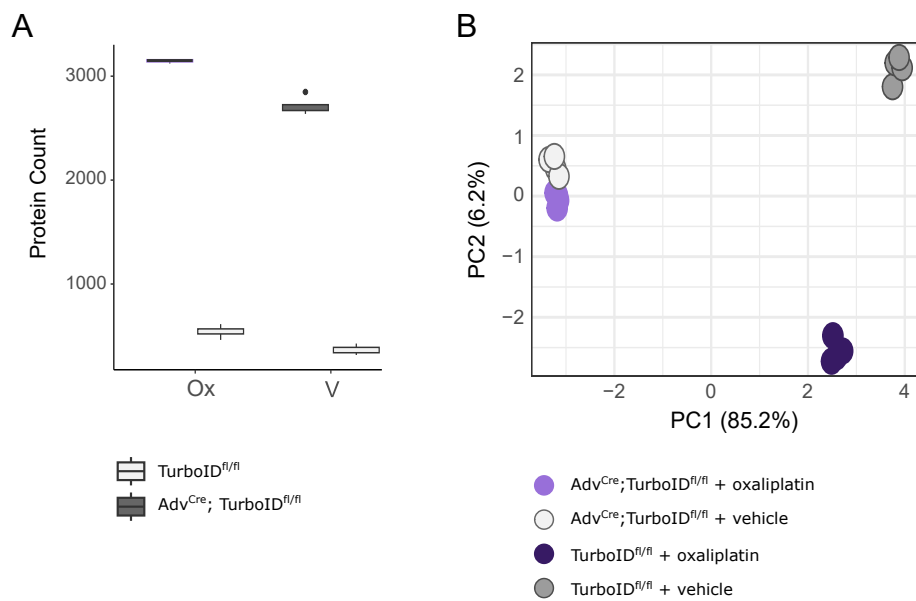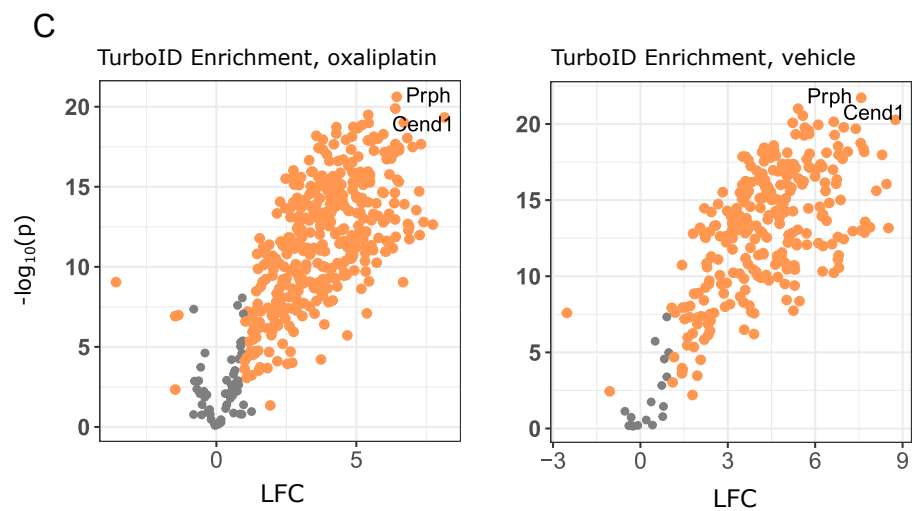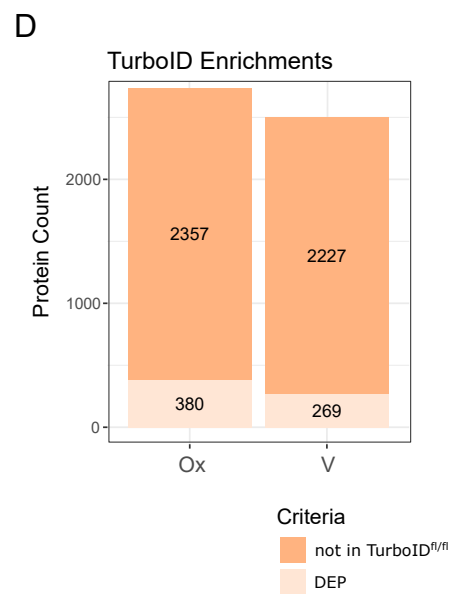

Supplement: Supplementary file 7 — Figure S7: Proteomics of DRG explants from AdvCre;TurboIDfl/fl and TurboIDfl/fl mice. A. Protein group counts across conditions. B. PCA across conditions. C. Differentially expressed proteins between AdvCre;TurboIDfl/fl and TurboIDfl/fl explant samples, for both vehicle and oxaliplatin‐treated explants. D. Numbers of enriched proteins, including both DEPs from C (AdvCre;TurboIDfl/fl vs TurboIDfl/fl), as well as proteins detected in AdvCre;TurboIDfl/fl but not TurboIDfl/fl controls (‘not in TurboIDfl/fl’), please see methods for details. LFC = log2 fold change. Ox = oxaliplatin. V = vehicle. [file EJP-30-0-s022.pdf]
