# Supplementary material for: Proximity Labelling Reveals the Compartmental Proteome of Murine Sensory Neurons
Source: Eur J Pain. 2026 Apr 26;30:e70277. doi: 10.1002/ejp.70277 (PMC13111903; doi:10.1002/ejp.70277)
Supplement: Supplementary file 24 — Table S17: Key resources. [file EJP-30-0-s020.docx]

**Supplemental Table 17**. Key Resources

| **Type** | **Designation** | **Source** | **Identifiers** | **Additional** |
| --- | --- | --- | --- | --- |
| Mouse strain | C57BL/6J | In house bred |  | Wild type, male |
| Mouse strain | Tg(Advillin-Cre)^+^ | Kind gift of Prof. Dr. Klaus-Armin Nave, Max Planck Institute for Multidisciplinary Sciences, Göttingen | (Zurborg et al., 2011) Tg(Avil-cre)1Phep MGI:5292346 |  |
| Mouse strain | TurboID^fl/fl^ | Current study | ROSA26^em1(TurboID)Bros^ |  |
| Chemical | Acetonitrile | Fisher Scientific | 10001334 |  |
| Chemical | Formic Acid | Fisher Scientific | 15658430 |  |
| Chemical | 10x PBS | Fisher Scientific | 11594516 |  |
| Chemical | Tris 1M | Accugene/Avantor | 51237 |  |
| Chemical | NaCl, 5M | Accugene/Avantor | 51202 |  |
| Chemical | Triton™ X-100 | Fisher Scientific | 10102913 |  |
| Chemical | Invitrogen™ SDS, 20% Solution, RNase-free | Fisher Scientific | AM9820 |  |
| Chemical | Biotin | Sigma-Aldrich | B4501-10G |  |
| Chemical | Oxaliplatin | Sigma-Aldrich | O9512-5MG |  |
| Chemical | Glycerol | Fisher Scientific | 10021083 |  |
| Chemical | Dithiothreitol 1M | Sigma-Aldrich | 43816 |  |
| Chemical | Acetone | Sigma-Aldrich | 1000201000 |  |
| Chemical | Ethanol | Sigma-Aldrich | 1117272500 |  |
| Chemical | Iodoacetamide | Sigma-Aldrich | I1149 |  |
| Chemical | Ammonium bicarbonate | Sigma-Aldrich | 09830-500G |  |
| Chemical | Water MS grade | Sigma-Aldrich | 1.15333.1000 |  |
| other | Trypsin/Lys-C | Promega | V5073 |  |
| other | rhβ-Nerve Growth Factor | R&D Systems | 256-GF |  |
| other | Gibco™ DMEM/F-12, GlutaMAX™ Supplement | Fisher Scientific | 31331028 |  |
| other | Sera-Mag SpeedBead beads | Cytiva | 65152105050250,  45152105050250 | 1:1 mix |
| other | Sera-Mag SpeedBead Blocked Streptavidin Particles | Cytiva | 21152104010150 |  |
| other | Complete protease inhibitor cocktail | Roche/ Merck | 58929700001 | Mix of protease inhibitors |
| other | Protein LoBind tube | Eppendorf | 0030108116, 0030108132 | Reagent tube |
| other | 4X Bolt™ LDS sample buffer | Fisher Scientific | B0007 |  |
| other | 10X Bolt™ Sample Reducing Agent | Fisher Scientific | B0009 |  |
| other | Bolt™ Bis-Tris Plus Mini Protein Gels, 4-12%, 1.0 mm | Fisher Scientific | NW04120BOX |  |
| other | Invitrogen™ iBlot™ 2 Transfer Stacks, PVDF, mini | Fisher Scientific | IB24002 |  |
| other | SlowFade™ Glass Soft-set Antifade Mountant, with DAPI | Fisher Scientific | S36920 |  |
| other | Aurora Series UHPLC column | IonOpticks | AUR2-25075C18A-CSI |  |
| other | Biopsy punch 4mm | Kai medical | 48401 |  |
| antibody | Streptavidin-AF680 | invitrogen | S21378 | 1:500 dilution |
| antibody | Mouse mc α-β-actin | Sigma-Aldrich | A1978-200UL | 1:500 dilution |
| antibody | Streptavidin-AF555 | Invitrogen | S32355 | 1:500 dilution |
| antibody | Rabbit α-NF200 | Sigma-Aldrich | N4142-25UL | 1:500 dilution |
| antibody | Chicken α-Peripherin | ABcam | AB39374 | 1:100 dilution |
| antibody | Donkey α-mouse AF790 | Invitrogen | A11371 | 1:8000 dilution |
| antibody | Donkey α-rabbit 488 | Invitrogen | A-21206 | 1:250 dilution |
| antibody | Donkey α-chicken 647 | Invitrogen | A78952 | 1:250 dilution |
| software, algorithm | DIA-NN | https://github.com/vdemichev/DiaNN |  | 1.8.2 |
| software, algorithm | R | https://www.r-project.org/ |  | 4.3.2 |
| software, algorithm | Mouse proteome | Uniprot | UP000000589 |  |
| software, algorithm | Cytoscape | https://cytoscape.org/ |  | 3.10.3 |
