# Supplementary material for: Proximity Labelling Reveals the Compartmental Proteome of Murine Sensory Neurons
Source: Eur J Pain. 2026 Apr 26;30:e70277. doi: 10.1002/ejp.70277 (PMC13111903; doi:10.1002/ejp.70277)
Supplement: Supplementary file 25 — Data S1: Supplemental methods: proximity labelling reveals the compartmental proteome of murine sensory neurons. [file EJP-30-0-s007.docx]

**Supplemental Tables + Methods:** **Proximity labelling reveals the compartmental proteome of murine sensory neurons**

Supplemental tables containing limma results are available display the following parameters: logFC (log fold change), AveExpr (average protein expression across samples, t (t-statistic), P.Value (P-value corresponding to the t-statistic), adj.P.Val (adjusted P-value; false discovery rate), B (log-odds of differential expression, used in the context of the empirical Bayes moderation of the standard errors).

| **Order** | **Content** |
| --- | --- |
| Supplemental Table 1 | expression matrix, TurboID (compartments) |
| Supplemental Table 2 | TurboID protein enrichment |
| Supplemental Table 3 | published comparisons |
| Supplemental Table 4 | sexual dimorphism |
| Supplemental Table 5 | GO compartment functions |
| Supplemental Table 6 | WGCNA functions |
| Supplemental Table 7 | PPI peripheral terminal functions |
| Supplemental Table 8 | PPI central terminal functions |
| Supplemental Table 9 | expression matrix, bulk (tissues) |
| Supplemental Table 10 | TurboID-enriched proteins |
| Supplemental Table 11 | expression matrix, explant (bulk) |
| Supplemental Table 12 | DEPs (bulk oxaliplatin) |
| Supplemental Table 13 | expression matrix, explant (TurboID) |
| Supplemental Table 14 | TurboID protein enrichment, explant |
| Supplemental Table 15 | DEPs (TurboID oxaliplatin) |
| Supplemental Table 16 | GO oxaliplatin treated-explant functions |
| Supplemental Table 17 | Key resources |

**Supplemental Methods: Proximity labelling reveals the compartmental proteome of murine sensory neurons**

*Generation of TurboID mutant mice using CRISPR/Cas9*

The conditional TurboID knock-in (cKI) mouse line was generated by site-directed CRISPR/Cas9 mutagenesis in the ROSA26 locus. Superovulated C57Bl6/J females were mated with C57Bl6/J males and fertilized eggs were collected. In-house produced CRISPR reagents (hCas9_mRNA, sgRNAs, preformed Cas9_sgRNA-RNP complexes and the homology-directed repair fragment (HDR fragment)) were microinjected into the pronucleus and the cytoplasm of pronuclear-stage zygotes using an Eppendorf Femtojet. Importantly, all nucleotide-based CRISPR/Cas9 reagents (sgRNAs and hCas9_mRNA) were used as RNA molecules and were not plasmid-encoded, reducing the probability of off-target effects due to the short lifetime of RNA-based reagents (Tycko et al., 2019; Doench et al., 2016). The TurboID-containing HDR fragment was generated by subcloning a V5-tag-TurboID sequence into the Ai14-ROSA26-based plasmid backbone (Madisen et al., 2010) and isolated as a linear DNA fragment by SacI/AgeI restriction enzyme double digestion. Two sgRNAs targeting the ROSA26 Locus were selected using the guide RNA selection tool CRISPOR (Haeussler et al, 2016; Concordet & Haeussler, 2018). The correct locus-specific insertion of the HDR fragment was confirmed by localization PCRs with primers upstream and downstream of the HDR sequence.

Breeding Strategy: The Adv^Cre^;TurboID^fl/fl^ line was regularly maintained on a C57Bl/6 background. For all experiments, Adv^Cre^;TurboID^fl/fl^ males were crossed to TurboID^fl/fl^ females to generate Adv^Cre^;TurboID^fl/fl^  experimental mice (of both sexes) as well as Adv^Cre^ - negative TurboID^fl/fl^  littermate controls (TurboID^fl/fl^, of both sexes). We employed a four-tier strategy to prevent and assess potential germline recombination in line with published recommendations (Luo et al., 2020): (1) all breedings were set up with Adv^Cre^ present in male but not female breeders, as suggested in another published Adv^Cre^ knock-in mouse line (<https://www.jax.org/strain/032536>); (2) Adv^Cre^ was always maintained in a heterozygous state; (3) all mice were genotyped; (4) our genotyping protocol included a primer that would detect a potential Cre-recombined allele, which has not been the case in any progeny generated. Thus, to the best of our knowledge, we did not observe germline recombination in Adv^Cre^;TurboID^fl/fl^ mice.

*Genotyping Strategy*

For TurboID genotyping, genomic DNA (gDNA) was isolated from tail biopsies using a genomic DNA isolation kit (Nexttec, #10.924). For the location PCRs, 20 μL reactions were prepared using 1 μL clean gDNA (15-80ng), 4 µL PrimerSet (4 pmol final each), 4 µL 5X Reaction Buffer (Finnzymes #F-524), 0.4 µL PhireHot-Start II Taq DNA Polymerase (Finnzymes #F-122L), 1 µL 10 mM dNTPs (Bioline #DM-515107), 4 µL Hi-Spec Additive (Bioline #HS-014101) and 5.6 µL H2O. Thermocycler parameters location-PCR Rosa26 left arm: 98°C for 5 min, (98 °C for 45 s, 64 °C for 30 s, 72 °C for 60 s) repeated for 34 cycles, final step at 72 °C for 10 min. Thermocycler parameters-location PCR Rosa26 right arm: 98°C for 5 min, (98 °C for 45 s, 68 °C for 30 s, 72 °C for 180 s) repeated for 34 cycles, final step at 72 °C for 10 min.

For the diagnostic routine genotyping PCR, 20 μL reactions were prepared using 1 μL clean gDNA (15-80ng), 4 µL PrimerSet (4 pmol final each), 4 µL 5X Reaction Buffer (Biozym #331620XL), 0.2 µL Hot-Start Taq DNA Polymerase (Biozym #331620XL), 1 µL 50 mM MgCl2 (AGCTLab stock) and 9.8 µL H2O. Thermocycler parameters: 96°C for 3 min, (94 °C for 30 s, 62 °C for 60 s, 72 °C for 60 s) repeated for 32 cycles, final step at 72 °C for 7 min.

*Rosa26 Locus sgRNAs*

- Sense Rosa26 protospacer-sgRNA1 sequence: 5‘-ACTCCAGTCTTTCTAGAAGA-3‘ (PAM = TGG)
- Antisense Rosa26 protospacer-sgRNA2 sequence: 5‘-CGCCCATCTTCTAGAAAGAC-3‘ (PAM = TGG)

**C57BL6J_Chrom6_Rosa26 target locus (insertion site):**


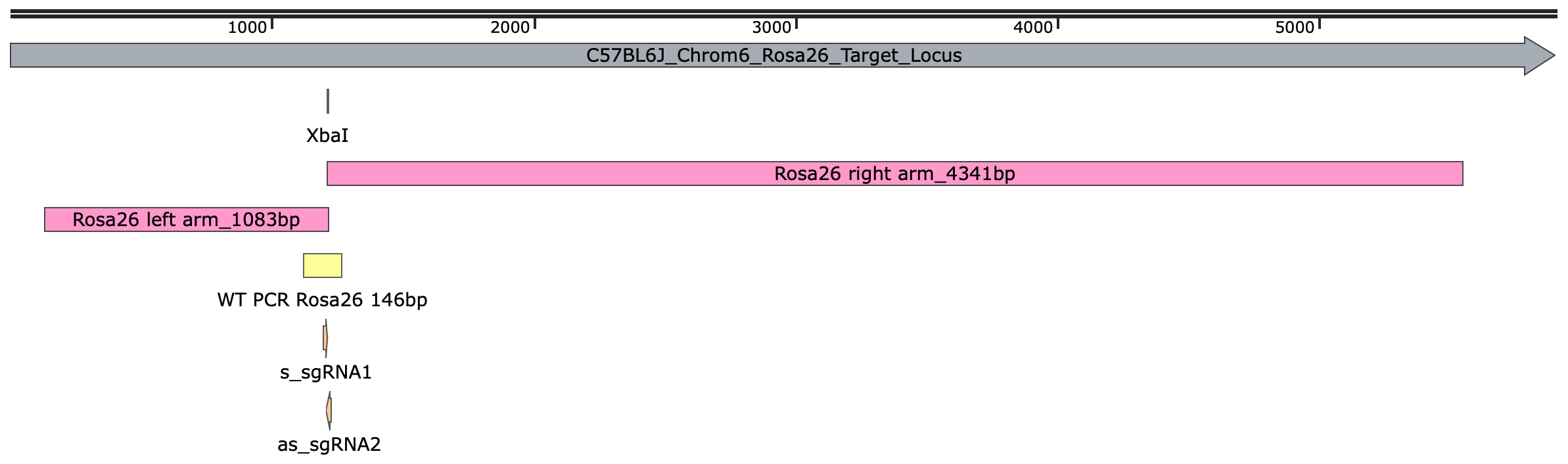


**Ai14-TurboID_Rosa26 HDR fragment targeting strategy (conditional knock-in)**


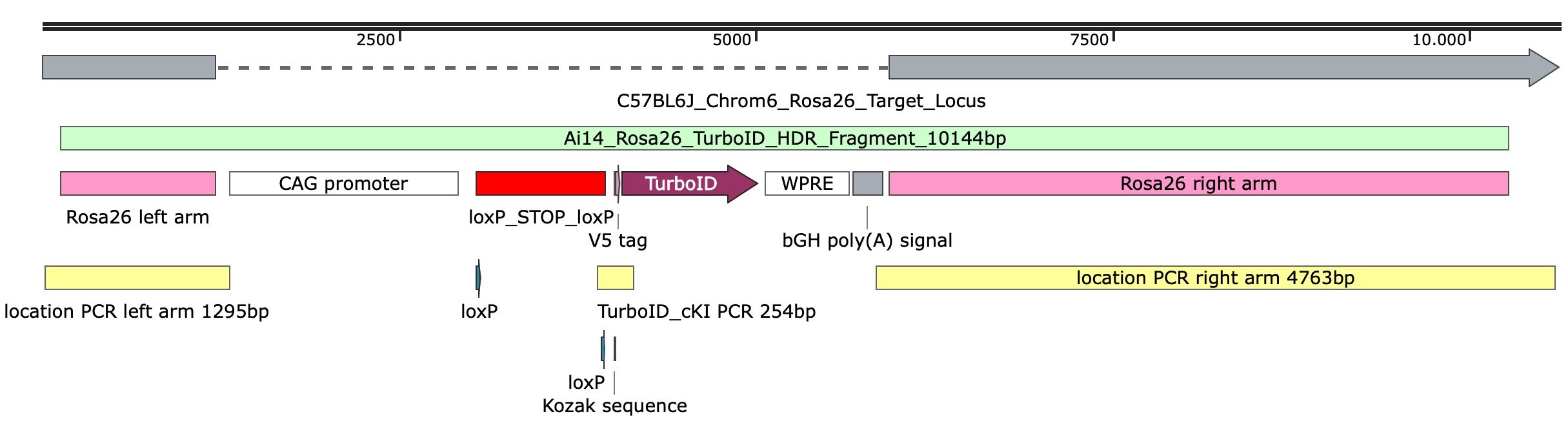


**Ai14-TurboID_Rosa26 Cre recombined (knock-in)**


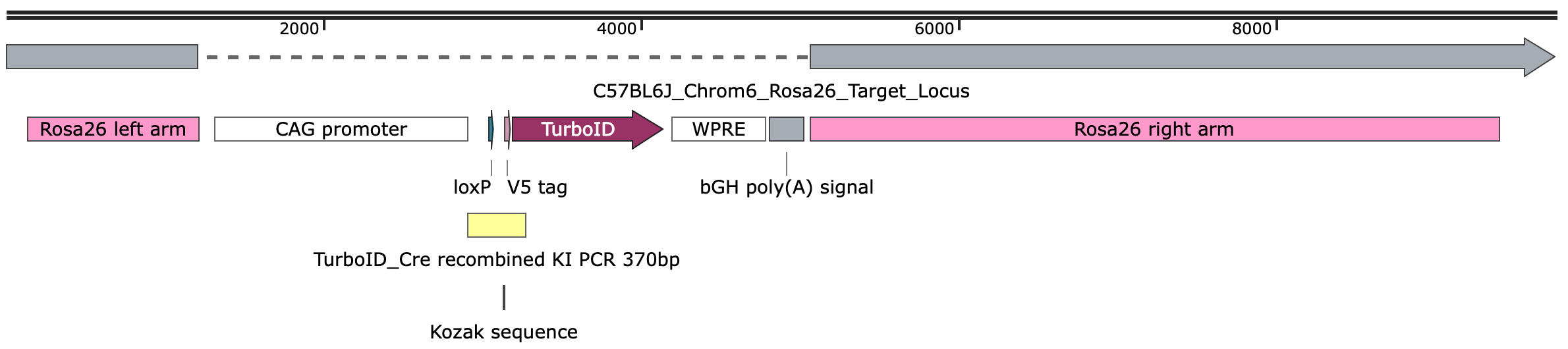


**SMethods, Figure1. Schematic representation of the target locus, the targeting strategy (HDR fragment) and the Cre recombination event.**

*Ai14-TurboID_Rosa26 HDR fragment and sequence annotations:*

Lower case letters = upstream and downstream HDR fragment ROSA26 sequences

Capital letters = HDR fragment after locus-specific insertion

Magenta = ROSA26 left- and right arm sequence

Blue = CAG-promoter

**Red BOLD** = loxP sites

**Underlined BOLD** = loxP flanked STOP cassette

**Green BOLD** = ATG Start- and TAA Stop codon

**Brown BOLD** = V5-tag

**Purple BOLD** = TurboID

**Black BOLD** = WPRE

Black underlined = bGH poly(A) signal

5’-ggaggctgccggggccgcctaaagaagaggctgtgctttggggctccggctcctcagagagcctcggctaggtaggggatcgggactctggcgggagggcggcttggtgcgtttgcggggatgggcggccGCGGCAGGCCCTCCGAGCGTGGTGGAGCCGTTCTGTGAGACAGCCGGGTACGAGTCGTGACGCTGGAAGGGGCAAGCGGGTGGTGGGCAGGAATGCGGTCCGCCCTGCAGCAACCGGAGGGGGAGGGAGAAGGGAGCGGAAAAGTCTCCACCGGACGCGGCCATGGCTCGGGGGGGGGGGGGCAGCGGAGGAGCGCTTCCGGCCGACGTCTCGTCGCTGATTGGCTTCTTTTCCTCCCGCCGTGTGTGAAAACACAAATGGCGTGTTTTGGTTGGCGTAAGGCGCCTGTCAGTTAACGGCAGCCGGAGTGCGCAGCCGCCGGCAGCCTCGCTCTGCCCACTGGGTGGGGCGGGAGGTAGGTGGGGTGAGGCGAGCTGGACGTGCGGGCGCGGTCGGCCTCTGGCGGGGCGGGGGAGGGGAGGGAGGGTCAGCGAAAGTAGCTCGCGCGCGAGCGGCCGCCCACCCTCCCCTTCCTCTGGGGGAGTCGTTTTACCCGCCGCCGGCCGGGCCTCGTCGTCTGATTGGCTCTCGGGGCCCAGAAAACTGGCCCTTGCCATTGGCTCGTGTTCGTGCAAGTTGAGTCCATCCGCCGGCCAGCGGGGGCGGCGAGGAGGCGCTCCCAGGTTCCGGCCCTCCCCTCGGCCCCGCGCCGCAGAGTCTGGCCGCGCGCCCCTGCGCAACGTGGCAGGAAGCGCGCGCTGGGGGCGGGGACGGGCAGTAGGGCTGAGCGGCTGCGGGGCGGGTGCAAGCACGTTTCCGACTTGAGTTGCCTCAAGAGGGGCGTGCTGAGCCAGACCTCCATCGCGCACTCCGGGGAGTGGAGGGAAGGAGCGAGGGCTCAGTTGGGCTGTTTTGGAGGCAGGAAGCACTTGCTCTCCCAAAGTCGCTCTGAGTTGTTATCAGTAAGGGAGCTGCAGTGGAGTAGGCGGGGAGAAGGCCGCACCCTTCTCCGGAGGGGGGAGGGGAGTGTTGCAATACCTTTCTGGGAGTTCTCTGCTGCCTCCTGGCTTCTGAGGACCGCCCTGGGCCTGGGAGAATCCCTTCCCCCTCTTCCCTCGTGATCTGCAACTCCAGTCTTTCTAGCCTTAATTAACCGTTTAAACAATTCTGCAGGAATCTAGTTATTAATAGTAATCAATTACGGGGTCATTAGTTCATAGCCCATATATGGAGTTCCGCGTTACATAACTTACGGTAAATGGCCCGCCTGGCTGACCGCCCAACGACCCCCGCCCATTGACGTCAATAATGACGTATGTTCCCATAGTAACGCCAATAGGGACTTTCCATTGACGTCAATGGGTGGAGTATTTACGGTAAACTGCCCACTTGGCAGTACATCAAGTGTATCATATGCCAAGTACGCCCCCTATTGACGTCAATGACGGTAAATGGCCCGCCTGGCATTATGCCCAGTACATGACCTTATGGGACTTTCCTACTTGGCAGTACATCTACGTATTAGTCATCGCTATTACCATGGTCGAGGTGAGCCCCACGTTCTGCTTCACTCTCCCCATCTCCCCCCCCTCCCCACCCCCAATTTTGTATTTATTTATTTTTTAATTATTTTGTGCAGCGATGGGGGCGGGGGGGGGGGGGGGGCGCGCGCCAGGCGGGGCGGGGCGGGGCGAGGGGCGGGGCGGGGCGAGGCGGAAAGGTGCGGCGGCAGCCAATCARAGCGGCGCGCTCCGAAAGTTTCCTTTTATGGCGAGGCGGCGGCGGCGGCGGCCCTATAAAAAGCGAAGCGCGCGGCGGGCGGGAGTCGCTGCGCGCTGCCTTCGCCCCGTGCCCCGYTCCGCCGCCGCCTCGCGCCGCCCGCCCCGGYTCTGACTGACCGCGTTACTCCCACAGGTGAGCGGGCGGGACGGCCCTTCTCCTCCGGGYTGTAATTAGCGCTTGGTTTAATGACGGYTTGTTTCTTTTCTGTGGYTGCGTGAAAGCCTTGAGGGGCTCCGGGAGGGCCCTTTGTGCGGGGGGAGCGGCTCGGGGGGTGCGTGCGTGTGTGTGTGCGTGGGGAGCGCCGCGTGCGGCTCCGCGCTGCCCGGCGGCTGTGAGCGCTGCGGGCGCGGCGCGGGGCTTTGTGCGCTCCGCAGTGTGCGCGAGGGGAGCGCGGCCGGGGGCGGTGCCCCGCGGTGCGGGGGGGGCTGCGAGGGGAACAAAGGCTGCGTGCGGGGTGTGTGCGTGGGGGGGTGAGCAGGGGGTGTGGGCGCGTCGGTCGGGCTGCAACCCCCCCTGCACCCCCCTCCCCGAGTTGCTGAGCACGGCCCGGCTTCGGGTGCGGGGCTCCGTACGGGGCGTGGCGCGGGGCTCGCCGTGCCGGGCGGGGGGTGGCGGCAGGTGGGGGTGCCGGGCGGGGCGGGGCCGCCTCGGGCCGGGGAGGGCTCGGGGGAGGGGCGCGGCGGCCCCCGGAGCGCCGGCGGCTGTCGAGGCGCGGCGAGCCGCAGCCATTGCCTTTTATGGTAATCGTGCGAGAGGGCGCAGGGACTTCCTTTGTCCCAAATCTGTGCGGAGCCGAAATCTGGGAGGCGCCGCCGCACCCCCTCTAGCGGGCGCGGGGCGAAGCGGTGCGGCGCCGGCAGGAAGGAAATGGGCGGGGAGGGCCTTCGTGCGTCGCCGCGCCGCCGTCCCCTTCTCCCTCTCCAGCCTCGGGGCTGTCCGCGGGGGGACGGCTGCCTTCGGGGGGGACGGGGCAGGGCGGGGTTCGGCTTCTGGCGTGTGACCGGCGGCTCTAGAGCCTCTGCTAACCATGTTCATGCCTTCTTCTTTTTCCTACAGCTCCTGGGCAACGTGCTGGTTATTGTGCTGTCTCATCATTTTGGCAAAGAATTGATTTGATACCGCGGGCCCTAAGAAGTTCCTATTCTCTAGAAAGTATAGGAACTTCGTCGACATTTAAATCATTTAAAT**ATAACTTCGTATAATGTATGCTATACGAAGTTATTCGCGATGAATAAATGAAAGCTTGCAGATCTGCGACTCTAGAGGATCTGCGACTCTAGAGGATCATAATCAGCCATACCACATTTGTAGAGGTTTTACTTGCTTTAAAAAACCTCCCACACCTCCCCCTGAACCTGAAACATAAAATGAATGCAATTGTTGTTGTTAACTTGTTTATTGCAGCTTATAATGGTTACAAATAAAGCAATAGCATCACAAATTTCACAAATAAAGCATTTTTTTCACTGCATTCTAGTTGTGGTTTGTCCAAACTCATCAATGTATCTTATCATGTCTGGATCTGCGACTCTAGAGGATCATAATCAGCCATACCACATTTGTAGAGGTTTTACTTGCTTTAAAAAACCTCCCACACCTCCCCCTGAACCTGAAACATAAAATGAATGCAATTGTTGTTGTTAACTTGTTTATTGCAGCTTATAATGGTTACAAATAAAGCAATAGCATCACAAATTTCACAAATAAAGCATTTTTTTCACTGCATTCTAGTTGTGGTTTGTCCAAACTCATCAATGTATCTTATCATGTCTGGATCTGCGACTCTAGAGGATCATAATCAGCCATACCACATTTGTAGAGGTTTTACTTGCTTTAAAAAACCTCCCACACCTCCCCCTGAACCTGAAACATAAAATGAATGCAATTGTTGTTGTTAACTTGTTTATTGCAGCTTATAATGGTTACAAATAAAGCAATAGCATCACAAATTTCACAAATAAAGCATTTTTTTCACTGCATTCTAGTTGTGGTTTGTCCAAACTCATCAATGTATCTTATCATGTCTGGATCCCCATCAAGCTGATCCGGAACCCTTAATATAACTTCGTATAATGTATGCTATACGAAGTTAT**TAGGTCCCTCGACCTGCAGCCCAAGCTAGATCGAATTCGGCCGGCCTTGTACGCGTGCCACC**ATGGGCAAGCCCATCCCCAACCCCCTGCTGGGCCTGGACAGCACC**GCTAGC**AAAGACAATACTGTGCCTCTGAAGCTGATCGCTCTCCTGGCTAATGGCGAGTTCCATAGTGGCGAACAGCTGGGAGAAACCCTGGGCATGTCCAGGGCCGCTATCAACAAGCACATTCAGACTCTGCGCGACTGGGGCGTGGACGTGTTCACCGTGCCCGGAAAGGGCTACTCTCTGCCCGAGCCTATCCCGCTGCTGAACGCTAAACAGATTCTGGGACAGCTGGACGGCGGGAGCGTGGCAGTCCTGCCTGTGGTCGACTCCACCAATCAGTACCTGCTGGATCGAATCGGCGAGCTGAAGAGTGGGGATGCTTGCATTGCAGAATATCAGCAGGCAGGGAGAGGAAGCAGAGGGAGGAAATGGTTCTCTCCTTTTGGAGCTAACCTGTACCTGAGTATGTTTTGGCGCCTGAAGCGGGGACCAGCAGCAATCGGCCTGGGCCCGGTCATCGGAATTGTCATGGCAGAAGCGCTGCGAAAGCTGGGAGCAGACAAGGTGCGAGTCAAATGGCCCAATGACCTGTATCTGCAGGATAGAAAGCTGGCAGGCATCCTGGTGGAGCTGGCCGGAATAACAGGCGATGCTGCACAGATCGTCATTGGCGCCGGGATTAACGTGGCTATGAGGCGCGTGGAGGAAAGCGTGGTCAATCAGGGCTGGATCACACTGCAGGAAGCAGGGATTAACCTGGACAGGAATACTCTGGCCGCTACGCTGATCCGAGAGCTGCGGGCAGCCCTGGAACTGTTCGAGCAGGAAGGCCTGGCTCCATATCTGCCACGGTGGGAGAAGCTGGATAACTTCATCAATAGACCCGTGAAGCTGATCATTGGGGACAAAGAGATTTTCGGGATTAGCCGGGGGATTGATAAACAGGGAGCCCTGCTGCTGGAACAGGACGGAGTTATCAAACCCTGGATGGGCGGAGAAATCAGTCTGCGGTCTGCCGAAAAGTAA**ACGCGTACAAGGCCGGCCCTGCAGGAATTCGATATCAAGCTTATCGAT**AATCAACCTCTGGATTACAAAATTTGTGAAAGATTGACTGGTATTCTTAACTATGTTGCTCCTTTTACGCTATGTGGATACGCTGCTTTAATGCCTTTGTATCATGCTATTGCTTCCCGTATGGCTTTCATTTTCTCCTCCTTGTATAAATCCTGGTTGCTGTCTCTTTATGAGGAGTTGTGGCCCGTTGTCAGGCAACGTGGCGTGGTGTGCACTGTGTTTGCTGACGCAACCCCCACTGGTTGGGGCATTGCCACCACCTGTCAGCTCCTTTCCGGGACTTTCGCTTTCCCCCTCCCTATTGCCACGGCGGAACTCATCGCCGCCTGCCTTGCCCGCTGCTGGACAGGGGCTCGGCTGTTGGGCACTGACAATTCCGTGGTGTTGTCGGGGAAATCATCGTCCTTTCCTTGGCTGCTCGCCTGTGTTGCCACCTGGATTCTGCGCGGGACGTCCTTCTGCTACGTCCCTTCGGCCCTCAATCCAGCGGACCTTCCTTCCCGCGGCCTGCTGCCGGCTCTGCGGCCTCTTCCGCGTCTTCGCCTTCGCCCTCAGACGAGTCGGATCTCCCTTTGGGCCGCCTCCCCGC**ATCGATACCGTCGACCTCGACCTCGACTGTGCCTTCTAGTTGCCAGCCATCTGTTGTTTGCCCCTCCCCCGTGCCTTCCTTGACCCTGGAAGGTGCCACTCCCACTGTCCTTTCCTAATAAAATGAGGAAATTGCATCGCATTGTCTGAGTAGGTGTCATTCTATTCTGGGGGGTGGGGTGGGGCAGGACAGCAAGGGGGAGGATTGGGAAGACAATGGCAGGCATGCTGGGGAACTAGTGGTGCCAGGGCGTGCCCTTGGGCTCCCCGGCGCGCCGGCTAGAAGATGGGCGGGAGTCTTCTGGGCAGGCTTAAAGGCTAACCTGGTGTGTGGGCGTTGTCCTGCAGGGGAATTGAACAGGTGTAAAATTGGAGGGACAAGACTTCCCACAGATTTTCGGTTTTGTCGGGAAGTTTTTTAATAGGGGCAAATAAGGAAAATGGGAGGATAGGTAGTCATCTGGGGTTTTATGCAGCAAAACTACAGGTTATTATTGCTTGTGATCCGCCTCGGAGTATTTTCCATCGAGGTAGATTAAAGACATGCTCACCCGAGTTTTATACTCTCCTGCTTGAGATCCTTACTACAGTATGAAATTACAGTGTCGCGAGTTAGACTATGTAAGCAGAATTTTAATCATTTTTAAAGAGCCCAGTACTTCATATCCATTTCTCCCGCTCCTTCTGCAGCCTTATCAAAAGGTATTTTAGAACACTCATTTTAGCCCCATTTTCATTTATTATACTGGCTTATCCAACCCCTAGACAGAGCATTGGCATTTTCCCTTTCCTGATCTTAGAAGTCTGATGACTCATGAAACCAGACAGATTAGTTACATACACCACAAATCGAGGCTGTAGCTGGGGCCTCAACACTGCAGTTCTTTTATAACTCCTTAGTACACTTTTTGTTGATCCTTTGCCTTGATCCTTAATTTTCAGTGTCTATCACCTCTCCCGTCAGGTGGTGTTCCACATTTGGGCCTATTCTCAGTCCAGGGAGTTTTACAACAATAGATGTATTGAGAATCCAACCTAAAGCTTAACTTTCCACTCCCATGAATGCCTCTCTCCTTTTTCTCCATTTATAAACTGAGCTATTAACCATTAATGGTTTCCAGGTGGATGTCTCCTCCCCCAATATTACCTGATGTATCTTACATATTGCCAGGCTGATATTTTAAGACATTAAAAGGTATATTTCATTATTGAGCCACATGGTATTGATTACTGCTTACTAAAATTTTGTCATTGTACACATCTGTAAAAGGTGGTTCCTTTTGGAATGCAAAGTTCAGGTGTTTGTTGTCTTTCCTGACCTAAGGTCTTGTGAGCTTGTATTTTTTCTATTTAAGCAGTGCTTTCTCTTGGACTGGCTTGACTCATGGCATTCTACACGTTATTGCTGGTCTAAATGTGATTTTGCCAAGCTTCTTCAGGACCTATAATTTTGCTTGACTTGTAGCCAAACACAAGTAAAATGATTAAGCAACAAATGTATTTGTGAAGCTTGGTTTTTAGGTTGTTGTGTTGTGTGTGCTTGTGCTCTATAATAATACTATCCAGGGGCTGGAGAGGTGGCTCGGAGTTCAAGAGCACAGACTGCTCTTCCAGAAGTCCTGAGTTCAATTCCCAGCAACCACATGGTGGCTCACAACCATCTGTAATGGGATCTGATGCCCTCTTCTGGTGTGTCTGAAGACCACAAGTGTATTCACATTAAATAAATAAATCCTCCTTCTTCTTCTTTTTTTTTTTTTTAAAGAGAATACTGTCTCCAGTAGAATTTACTGAAGTAATGAAATACTTTGTGTTTGTTCCAATATGGTAGCCAATAATCAAATTACTCTTTAAGCACTGGAAATGTTACCAAGGAACTAATTTTTATTTGAAGTGTAACTGTGGACAGAGGAGCCATAACTGCAGACTTGTGGGATACAGAAGACCAATGCAGACTTTAATGTCTTTTCTCTTACACTAAGCAATAAAGAAATAAAAATTGAACTTCTAGTATCCTATTTGTTTAAACTGCTAGCTTTACTTAACTTTTGTGCTTCATCTATACAAAGCTGAAAGCTAAGTCTGCAGCCATTACTAAACATGAAAGCAAGTAATGATAATTTTGGATTTCAAAAATGTAGGGCCAGAGTTTAGCCAGCCAGTGGTGGTGCTTGCCTTTATGCCTTTAATCCCAGCACTCTGGAGGCAGAGACAGGCAGATCTCTGAGTTTGAGCCCAGCCTGGTCTACACATCAAGTTCTATCTAGGATAGCCAGGAATACACACAGAAACCCTGTTGGGGAGGGGGGCTCTGAGATTTCATAAAATTATAATTGAAGCATTCCCTAATGAGCCACTATGGATGTGGCTAAATCCGTCTACCTTTCTGATGAGATTTGGGTATTATTTTTTCTGTCTCTGCTGTTGGTTGGGTCTTTTGACACTGTGGGCTTTCTTTAAAGCCTCCTTCCTGCCATGTGGTCTCTTGTTTGCTACTAACTTCCCATGGCTTAAATGGCATGGCTTTTTGCCTTCTAAGGGCAGCTGCTGAGATTTGCAGCCTGATTTCCAGGGTGGGGTTGGGAAATCTTTCAAACACTAAAATTGTCCTTTAATTTTTTTTTTAAAAAATGGGTTATATAATAAACCTCATAAAATAGTTATGAGGAGTGAGGTGGACTAATATTAAATGAGTCCCTCCCCTATAAAAGAGCTATTAAGGCTTTTTGTCTTATACTTAACTTTTTTTTTAAATGTGGTATCTTTAGAACCAAGGGTCTTAGAGTTTTAGTATACAGAAACTGTTGCATCGCTTAATCAGATTTTCTAGTTTCAAATCCAGAGAATCCAAATTCTTCACAGCCAAAGTCAAATTAAGAATTTCTGACTTTTAATGTTAATTTGCTTACTGTGAATATAAAAATGATAGCTTTTCCTGAGGCAGGGTCTCACTATGTATCTCTGCCTGATCTGCAACAAGATATGTAGACTAAAGTTCTGCCTGCTTTTGTCTCCTGAATACTAAGGTTAAAATGTAGTAATACTTTTGGAACTTGCAGGTCAGATTCTTTTATAGGGGACACACTAAGGGAGCTTGGGTGATAGTTGGTAAAATGTGTTTCAAGTGATGAAAACTTGAATTATTATCACCGCAACCTACTTTTTAAAAAAAAAAGCCAGGCCTGTTAGAGCATGCTTAAGGGATCCCTAGGACTTGCTGAGCACACAAGAGTAGTTACTTGGCAGGCTCCTGGTGAGAGCATATTTCAAAAAACAAGGCAGACAACCAAGAAACTACAGTTAAGGTTACCTGTCTTTAAACCATCTGCATATACACAGGGATATTAAAATATTCCAAATAATATTTCATTCAAGTTTTCCCCCATCAAATTGGGACATGGATTTCTCCGGTGAATAGGCAGAGTTGGAAACTAAACAAATGTTGGTTTTGTGATTTGTGAAATTGTTTTCAAGTGATAGTTAAAGCCCATGAGATACAGAACAAAGCTGCTATTTCGAGGTCTCTTGGTTTATACTCAGAAGCACTTCTTTGGGTTTCCCTGCACTATCCTGATCATGTGCTAGGCCTACCTTAGGCTGATTGTTGTTCAAATAAACTTAAGTTTCCTGTCAGGTGATGTCATATGATTTCATATATCAAGGCAAAACATGTTATATATGTTAAACATTTGTACTTAATGTGAAAGTTAGGTCTTTGTGGGTTTGATTTTTAATTTTCAAAACCTGAGCTAAATAAGTCATTTTTACATGTCTTACATTTGGTGGAATTGTATAATTGTGGTTTGCAGGCAAGACTCTCTGACCTAGTAACCCTACCTATAGAGCACTTTGCTGGGTCACAAGTCTAGGAGTCAAGCATTTCACCTTGAAGTTGAGACGTTTTGTTAGTGTATACTAGTTTATATGTTGGAGGACATGTTTATCCAGAAGATATTCAGGACTATTTTTGACTGGGCTAAGGAATTGATTCTGATTAGCACTGTTAGTGAGCATTGAGTGGCCTTTAGGCTTGAATTGGAGTCACTTGTATATCTCAAATAATGCTGGCCTTTTTTAAAAAGCCCTTGTTCTTTATCACCCTGTTTTCTACATAATTTTTGTTCAAAGAAATACTTGTTTGGATCTCCTTTTGACAACAATAGCATGTTTTCAAGCCATATTTTTTTTCCTTTTTTTTTTTTTTTTTGGTTTTTCGAGACAGGGTTTCTCTGTATAGCCCTGGCTGTCCTGGAACTCACTTTGTAGACCAGGCTGGCCTCGAACTCAGAAATCCGCCTGCCTCTGCCTCCTGAGTGCCGGGATTAAAGGCGTGCACCACCACGCCTGGCTAAGTTGGATATTTTGTTATATAACTATAACCAATACTAACTCCACTGGGTGGATTTTTAATTCAGTCAGTAGTCTTAAGTGGTCTTTATTGGCCCTTCATTAAAATCTACTGTTCACTCTAACAGAGGCTGTTGGTACTAGTGGCACTTAAGCAACTTCCTACGGATATACTAGCAGATTAAGGGTCAGGGATAGAAACTAGTCTAGCGTTTTGTATACCTACCAGCTTTATACTACCTTGTTCTGATAGAAATATTTCAGGACATCTAGagtgtactataaggttgatggtaagcttataaggaacttgaaagtggagtaactactccatttctctgaggggagaattaaaatttttgaccaagtgttgttgagccactgagaatggtctcagaacataacttcttaaggaaccttcccagattgccctcaacactgcaccacatttggtcctgcttgaacattgccatggctcttaaagtcttaattaagaatattaattgtgtaattattgtttttcctcctttagatcattccttgaggacaggacagtgcttgtttaaggctatatttctgctgtctgagcagcaacaggtcttcgagatcaacatgatgttcat-3’

**SMethods, Sequence.** Ai14-TurboID_Rosa26 HDR fragment and sequence annotations.

**SMethods, Table. Primer sequence and fragment size information**

| **Name** | **ID** | **Sequence (5' → 3')** | **mer** | **Orientation** | **Target Region** | **Fragment Size** |
| --- | --- | --- | --- | --- | --- | --- |
| Location Rosa26 Left Arm | 29194 | CGCCTAAAGAAGAGGCTGTG | 20 | Sense | Upstream ROSA26 left-arm | 1295 bp (Location Left Arm) |
|  | 7604 | CGCGGAACTCCATATATGGGCT | 22 | Anti-sense | 5’-End-CAG-promoter |  |
| Location Rosa26 Right Arm | 2736 | GCAGGACAGCAAGGGGGAGGAT | 22 | Sense | bGH poly(A) signal | 4736 bp (Location Right arm) |
|  | 36502 | GACCTGTTGCTGCTCAGACAGC | 22 | Anti-sense | Downstream Rosa26 right arm |  |
| **Diagnostic Routine Genotyping PCR** | 14025 | CTCTGCTGCCTCCTGGCTTCT | 21 | Sense | ROSA26 upstream insertion site | 146 bp (WT) |
|  | 24229 | CGCCCACACACCAGGTTAG | 19 | Anti-sense | ROSA26 downstream insertion site |  |
|  | 30545 | AAGCTGATCCGGAACCCTTAA | 21 | Sense | Upstream loxP2 | 254 bp (cKI) 370 bp (Cre recombined) |
|  | 26512 | CTACAGCTCCTGGGCAACG | 19 | Sense | upstream loxP1 (3’-End CAG promoter) |  |
|  | 38281 | CCAGGGTTTCTCCCAGCTGT | 20 | Anti-sense | TurboID |  |
